# Left atrial size and risk of recurrent ischemic stroke in a Chinese population

**DOI:** 10.1002/brb3.702

**Published:** 2017-04-12

**Authors:** Jie Xue, Yuanshao Lin, Wensi Huang, Xiaoli Chen, Qian Li, Zhengyi Cai, Wanli Zhang, Yuansheng Ye, Bei Shao

**Affiliations:** ^1^ Department of NeurologyThe First Affiliated Hospital of Wenzhou Medical UniversityWenzhouChina; ^2^ Department of NeurologyThe People's Hospital of PingyangWenzhouChina; ^3^ Department of Internal MedicineThe Third People's Hospital of YueqingYueqingChina

**Keywords:** echocardiography, left atrial size, predictor, recurrent ischemic stroke

## Abstract

**Background:**

Although a number of studies have reported the role of an increased left atrial (LA) size on stroke, limited data are collected about the relationship between LA enlargement and recurrent ischemic stroke in the Chinese population. Our aim was to assess the association of LA size with the risk of stroke recurrence, particularly with recurrent cardioembolic or cryptogenic stroke in ischemic stroke patients.

**Methods:**

The study recruited 313 consecutive patients with acute first‐ever ischemic stroke. Echocardiographic LA diameter was measured and indexed by height and body surface area separately. The endpoint was recurrent ischemic stroke. Cox proportional hazard models were used to examine the association of LA size with total recurrent ischemic stroke and recurrent cardioembolic or cryptogenic stroke while adjusting for baseline demographics characteristics, clinical factors, echocardiographic left ventricular ejection fraction, and medication.

**Results:**

Over a median follow‐up period of 1.63 years, 47 recurrent ischemic strokes (21 were cardioembolic or cryptogenic) occurred. In a multivariate model adjusted for potential confounders, compared with the bottom tertiles of LA diameter indexed to height (LA diameter/H), the top tertile of LA diameter/H was significantly associated with the total recurrent ischemic stroke (adjusted HR 3.610, 95% CI 1.870–6.967, *p *< .001) and the composite of recurrent cardioembolic or cryptogenic stroke (adjusted HR 5.673, 95% CI 1.780–18.084, *p *=* *.003). Results were similar when LA diameter indexed to body surface area (LA diameter/BSA) was involved in the analysis.

**Conclusion:**

LA size is an independent predictor of total recurrent ischemic stroke and the composite of recurrent cardioembolic or cryptogenic stroke.

## 1 Introduction

Stroke is the second leading cause of death in the world (Johnston, Mendis, & Mathers, 2009) but is the first cause of death and adult disability in China (Liu, Wang, Wong, & Wang, 2011). It is characterized by high morbidity, high mortality, and a high recurrence rate therefore posing a significant burden on health resources. Ischemic stroke is the most common type of stroke that accounts for almost 80–85% of all stroke cases and is brought about by cutting off cerebral blood flow and thereby oxygen to the brain (Allen & Bayraktutan, 2009). Although a variety of risk factors have been established for ischemic stroke, about 25% of stroke cases are considered cryptogenic (Fonseca & Ferro, 2015). As many of cryptogenic strokes are thought to arise from embolisms, it was thus defined as embolic stroke of an undetermined source (Hart et al., 2014); about 10% of patients with first‐ever ischemic stroke met this criteria (Ntaios et al., 2015).

As one of the four chambers of the heart, the left atrium plays a major role in cardiac physiology through a variety of mechanical functions to adjust left ventricular (LV) filling and release natriuretic peptides that senses blood volume (Abhayaratna et al., 2006; Leung, Boyd, Ng, Chi, & Thomas, 2008). Left atrial (LA) size increases in response to two main pathophysiologic conditions: pressure and volume overload (Leung et al., 2008). As an indicator of diastolic burden, LA enlargement is proven to be correlated with higher risk of atrial fibrillation (AF; Psaty et al., 1997; Tsang et al., 2001; Vaziri, Larson, Benjamin, & Levy, 1994), cardiovascular events (Gardin et al., 2001; Kizer et al., 2006), and all‐cause mortality (Bouzas‐Mosquera et al., 2011; Nagarajarao et al., 2008). A number of studies performed in the general population have evaluated the relationship between left atrial size and stroke, but the conclusions vary (Barnes et al., 2004; Benjamin, D'Agostino, Belanger, Wolf, & Levy, 1995; Bouzas‐Mosquera et al., 2011; Di Tullio, Sacco, Sciacca, & Homma, 1999; Gardin et al., 2001; Kizer et al., 2006; Nagarajarao et al., 2008). Some authors (Barnes et al., 2004; Nagarajarao et al., 2008) supported the association between LA enlargement and stroke while others (Gardin et al., 2001; Kizer et al., 2006) oppose it, and some found that the association was influenced by gender (Benjamin et al., 1995; Bouzas‐Mosquera et al., 2011; Di Tullio et al., 1999). However, the relationship between LA size and recurrent ischemic stroke was less reported. In a recent prospective study, moderate to severe LA enlargement was found to be associated with the risk of recurrent cardioembolic and cryptogenic stroke, suggesting that these two stroke subtypes may share a common embolic mechanism (Yaghi et al., 2015). If true, we would expect that LA enlargement results in thrombosis in the left atrium, then causes embolic stroke (cardioembolic and cryptogenic stroke). Thus, further studies are still necessary to provide more useful data for the better understanding of this relationship.

The aim of this study was to investigate the association between LA size and recurrent ischemic stroke in patients with acute ischemic stroke. We hypothesized that echocardiographic LA size would be associated with recurrent ischemic stroke, especially with cardioembolic and cryptogenic stroke.

## 2 Methods

### 2.1 Study population

This is a hospital‐based observational study performed in the Department of Neurology at the First Affiliated Hospital of Wenzhou Medical University. A total of 313 consecutive patients with first‐ever acute ischemic stroke were enrolled in the present study between December 2013 and July 2015. Inclusion criteria included (1) age ≥18 years; (2) a diagnosis of acute ischemic stroke; (3) the willingness to provide informed consent. Patients were excluded if they had any previous history of ischemic stroke or transient ischemic attack as well as the absence of a transthoracic echocardiogram. Stroke was defined as the first symptomatic occurrence of fatal and nonfatal ischemic stroke according to the recommendations from World Health Organization (WHO, 1989). The ischemic stroke etiologic subtype was determined according to the TOAST (Trial of Org 10172 in Acute Stroke Treatment) criteria (Adams et al., 1993). The study was approved by the ethics committee of the First Affiliated Hospital of Wenzhou Medical University. All patient or their relatives signed the informed consent before inclusion in this study.

### 2.2 Clinical data

Demographic, clinical, echocardiographic data and medication were collected at baseline for all patients. Hypertension was defined as systolic blood pressure (BP) of 140 mmHg, diastolic BP of 90 mmHg, the reported use of antihypertensive medications, or a history of diagnosed hypertension. Diabetes mellitus was defined as fasting serum glucose ≥126 mg/dl (7 mmol/L), nonfasting glucose ≥ 200 mg/dl (11.1 mmol/L), use of diabetic medications, or a previously established diabetic diagnosis. Hyperlipidemia was diagnosed as LDL‐cholesterol level ≥4.1 mmol/L, total cholesterol level ≥6.2 mmol/L, or use of lipid‐lowering agents after being diagnosed with hyperlipidemia. A history of AF was defined as atrial fibrillation recorded at the time of the electrocardiography or any previously known episode of atrial fibrillation. Smoking was defined as current or former cigarette smoking. Medications including the use of antiplatelet or anticoagulation agents were recorded at the time of discharge.

### 2.3 Echocardiography measurements

Transthoracic echocardiography was performed in the left lateral decubitus position using standard imaging planes, according to the recommendations of the American Society of Echocardiography (Sahn, DeMaria, Kisslo, & Weyman, 1978). Left atrial diameter was measured using two‐dimensional echocardiography, from the posterior aortic wall to the posterior left atrial wall, in the parastemal long‐axis view at the end‐ventricular systole. LA dimensions are the most commonly used echocardiographic parameter for LA size, but they vary between individuals. In this study, LA diameter was measured and indexed to height (LA diameter/H; Nagarajarao et al., 2008) and to body surface area (LA diameter/BSA; Di Tullio et al., 1999) separately. Left ventricular (LV) ejection fraction was estimated using the Teichholz formula or the Simpson rule. The data of the echocardiographic measurements were blinded to follow‐up visits.

### 2.4 Follow‐up

Patients were followed‐up in our hospital outpatient department or by telephone interviews. The endpoint of this study was recurrent ischemic stroke. The occurrence of recurrent ischemic stroke was recorded if this diagnosis was listed in the discharge summary from electronic medical records in our hospital during follow‐up visits. For recurrent stroke patients who were hospitalized elsewhere, the data were collected in the outpatient department or were self‐reported (4%). The primary outcome was total recurrent ischemic stroke, and the secondary outcome was the combined subtypes of recurrent cardioembolic and cryptogenic stroke. The follow‐up period was defined as the time from the date of admission to the date of the recurrent ischemic stroke or to June 30, 2016, if no ischemic stroke event was detected. Six patients were lost, and seven patients were deceased during the follow‐up.

### 2.5 Statistical analysis

Data are presented as mean ± *SD* or median (interquartile range, IQR) for continuous variables and percentages for categorical variables. Differences between mean values were assessed using the unpaired *t* test or one‐way analysis of variance (ANOVA) where appropriate. Kruskal–Wallis test was used for the comparison of non‐normally distributed variables. Comparison between categorical variables was assessed by the chi‐square test. A two‐sided probability value <0.05 was used to assess statistical significance. In the absence of any established standards for LA diameter/H, demographics and clinical characteristics were examined by tertile of LA diameter/H; thereafter, the bottom two tertiles (range: 15.6–25.3 mm/m) were grouped into one category and compared with the top tertile (range: 25.4–39.0 mm/m). The same grouping methods were used based on tertile of LA diameter/BSA. The primary predictor was LA diameter/H categories, and the secondary predictor was LA diameter/BSA. These two LA indexes were also used in the analysis as continuous variables. The recurrent stroke rate was expressed as the number of recurrent ischemic stroke per 100 patient‐years. Cox proportional hazard models were established to evaluate the association of the top tertile of LA size with the risk of recurrent ischemic stroke. Hazard ratios (HRs) and 95% confidence intervals (95% CI) were estimated. Covariates considered for inclusion in the multivariate analyses included demographics characteristics (age, sex), clinical risk factors (hypertension, diabetes mellitus, hypercholesterolemia, smoking, AF), echocardiographic LV ejection fraction, and the use of antiplatelet or anticoagulation agents. Analyses were performed with the SPSS 22 software package for Windows (SPSS Inc., Chicago, IL, USA, RRID:SCR_002865).

## 3 Results

### 3.1 Baseline characteristics

Overall, 313 consecutive patients with a first‐ever ischemic stroke fulfilled the study criteria. The median age of the patients was 64.0 years (IQR 56.0–70.0), and 190 patients (60.7%) were male. Table 1 shows the baseline demographics and clinical characteristics of study population by LA size categories. The patients in the top tertile of LA diameter/H (range: 25.3–39.0 mm/m) were older, less likely to smoke and had a higher incidence of AF. The patients in the top tertile of LA diameter/BSA (range: 25.1–40.3 mm/m^2^) were older, more likely women, less likely to smoke, and had a higher incidence of AF.

**Table 1:** Baseline demographics and clinical characteristics based on the Left Atrial size categories

Characteristics	Total (313)	LA diameter/H			LA diameter/BSA		
		Bottom two tertiles (15.6–25.3 mm/m) (212)	Top tertile (25.4–39.0 mm/m) (102)	*p* value	Bottom two tertiles (15.8–25.0 mm/m^2^) (209)	Top tertile (25.1–40.3 mm/m^2^) (104)	*p* value
Age, year, median (IQR)	64.0 (56.0–70.0)	62.0 (55.0–69.0)	65.5 (59.0–71.3)	.001	62.0 (54.0–68.0)	66.0 (62.0–73.0)	<.001
Gender (male)	190 (60.7)	135 (64.0)	55 (53.9)	.088	143 (68.4)	47 (45.2)	<.001
Hypertension	249 (79.6)	164 (77.7)	85 (83.3)	.249	162 (77.5)	87 (83.7)	.204
Diabetes	117 (37.4)	79 (37.4)	38 (37.3)	.975	82 (39.2)	35 (33.7)	.336
Hypercholesterolemia	105 (33.5)	71 (33.6)	34 (33.3)	.956	68 (32.5)	37 (35.6)	.591
AF	30 (9.6)	2 (0.9)	28 (27.5)	<.001	4 (1.9)	26 (25.0)	<.001
History of smoking	116 (37.1)	90 (42.7)	26 (25.5)	.003	91 (43.5)	25 (24.0)	.001
Antiplatelet agents	285 (91.1)	206 (97.6)	79 (77.5)	<.001	203 (97.1)	82 (78.8)	<.001
Anticoagulation agents	22 (7.0)	1 (0.5)	21 (20.6)	<.001	4 (1.9)	18 (17.3)	<.001
LA diameter/H, mm/m median (IQR)	24.2 (22.1–26.5)	23.1 (21.5–24.3)	27.3 (26.5–29.3)	<.001	23.1 (21.5–24.4)	27.2 (26.0–29.2)	<.001
LA diameter/BSA, mm/m^2^ median (IQR)	23.7 (21.8–26.0)	22.6 (21.2–24.1)	27.0 (25.2–29.3)	<.001	22.5 (21.2–23.8)	2.69 (25.5–29.2)	<.001
LV ejection fraction (mean ± *SD*)	65.4 ± 6.2	65.7 ± 5.8	64.8 ± 7.1	.236	65.7 ± 5.8	64.8 ± 7.0	.236
Stroke etiologic subtypes							
Atherosclerotic	191 (61.0)	143 (67.8)	48 (47.1)	<.001	136 (65.1)	55 (52.9)	<.001
Cardioembolic	32 (10.2)	4 (1.9)	28 (27.5)		6 (2.9)	26 (25.0)	
Lacunar	46 (14.7)	34 (16.1)	12 (11.8)		37 (17.7)	9 (8.7)	
Cryptogenic	41 (13.1)	27 (12.8)	14 (13.1)		28 (13.4)	13 (12.5)	
Follow‐up time, year, median (IQR)	1.63 (1.28–1.98)	1.65 (1.41–2.04)	1.53 (0.97–1.91)	.001	1.65 (1.42–2.04)	1.50 (0.97–1.91)	.001

AF, atrial fibrillation; IQR, interquartile range; LA diameter/H, left atrial diameter/height; LA diameter/BSA, left atrial diameter/ body surface area; LV, left ventricular; SD, standard deviation.

Data are presented as means (±*SD*) and medians (IQR) or as number (percentage).

Over a median of 1.63 years (IQR 1.28–1.98) of follow‐up, 47 (15.0%) incident recurrent ischemic strokes occurred. Table 2 shows the baseline demographics and clinical characteristics of study population related to recurrent ischemic stroke. The patients with recurrent ischemic stroke were older, had a higher incidence of AF, and less use of antiplatelet agents. Compared to the patients without recurrent stroke, LA diameter/H was significantly higher in patients with recurrent ischemic stroke [26.3 mm/m (IQR 23.4–28.1) *vs*. 24.1 mm/m (IQR 22.0–25.9), *p *= .004]. Similarly, LA diameter/BSA was also found higher in the patients with recurrent ischemic stroke [25.4 mm/m^2^ (IQR 22.7–27.8) vs. 23.6 mm/m^2^ (IQR 21.7–25.6), *p *= .003].

**Table 2:** Characteristics of patients with and without recurrent ischemic stroke

Characteristic	Total (313)	No recurrent ischemic stroke (266)	Recurrent ischemic stroke (47)	*p* value
Age, year, median (IQR)	64.0 (56.0–70.0)	63.0 (56.0–69.3)	66.0 (59.0–75.0)	.003
Gender (male)	190 (60.7)	157 (59.0)	33 (70.2)	.148
Hypertension	249 (79.6)	212 (79.7)	37 (78.7)	.878
Diabetes	117 (37.4)	102 (38.3)	15 (31.9)	.401
Hypercholesterolemia	105 (33.5)	86 (32.3)	19 (40.4)	.279
AF	30 (9.6)	21 (7.9)	9 (19.1)	.032
History of smoking	116 (37.1)	99 (37.2)	17 (36.2)	.891
Antiplatelet agents	285 (91.1)	248 (93.2)	37 (78.7)	.003
Anticoagulation agents	22 (7.0)	16 (6.0)	6 (12.8)	.174
LA diameter/H, mm/m median (IQR)	24.2 (22.1–26.5)	24.1 (22.0–25.9)	26.3 (23.4–28.1)	.004
LA diameter/BSA, mm/m^2^ median (IQR)	23.7 (21.8–26.0)	23.6 (21.7–25.6)	25.4 (22.7–27.8)	.003
LV ejection fraction (mean ± *SD*)	65.4 ± 6.2	65.3 ± 6.3	65.8 ± 6.0	.587
Follow‐up time, year				
Median (IQR)	1.63 (1.28–1.98)	1.69 (1.45–2.04)	0.52 (0.29–1.15)	<.001

AF, atrial fibrillation; IQR, interquartile range; LA diameter/H, left atrial diameter/height; LA diameter/BSA, left atrial diameter/ body surface area; LV, left ventricular; SD, standard deviation.

Data are presented as means (±*SD*) and medians (IQR) or as number (percentage).

### 3.2 Association between LA size and recurrent ischemic stroke of any subtype

**Figure 1:**
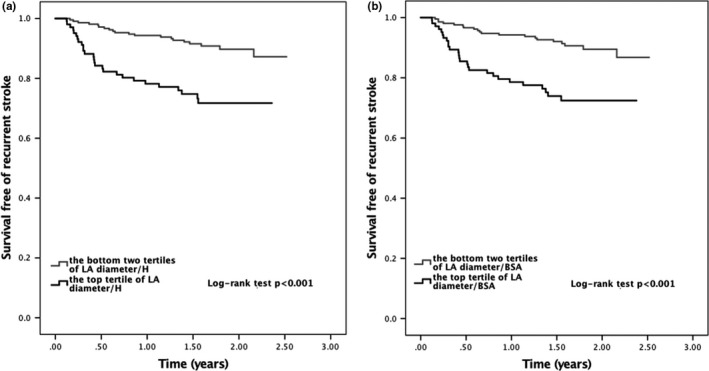
Survival curves free of total recurrent ischemic stroke according to the left atrial diameter/height (a) and left atrial diameter/body surface area (b)

Over the follow‐up period for recurrent ischemic stroke, the total recurrent stroke rate was 9.21 per 100 person‐years. The incidence rate of total recurrent ischemic stroke in the top tertile of LA diameter/H was 17.3 per 100 person‐years, which almost tripled the rates in the lower two tertiles. LA diameter/H was associated with recurrent ischemic stroke in the unadjusted model (unadjusted HR 3.310, 95% CI 1.854–5.912, *p *< .001) and in the multivariable model adjusting for age, sex, hypertension, diabetes mellitus, hypercholesterolemia, atrial fibrillation, smoking, LV ejection fraction, and the use of antiplatelet and anticoagulation agents (adjusted HR 3.610, 95% CI 1.870–6.967, *p *< .001; Table 3). LA diameter/H as a continuous variable was also associated with total recurrent ischemic stroke (unadjusted HR 1.138 per 1 mm/m change in LA diameter/H, 95% CI 1.056–1.226, *p *= .001; adjusted HR 1.142 per 1 mm/m change in LA diameter/H, 95% CI 1.031–1.265, *p *= .011). Similar results were found when LA diameter/BSA was used for analysis. Kaplan–Meier survival analysis is shown in Figure 1.

**Table 3:** Association between LA size and recurrent ischemic stroke of any subtype

Left atrial size	No. of recurrent ischemic stroke	Unadjusted HR (95% CI)	Adjusted HR (95% CI)
LA diameter/H			
Bottom two tertiles	20 (5.7)	Ref	Ref
Top tertile	27 (17.3)	3.310 (1.854–5.912, *p *< .001)	3.610 (1.870–6.967, *p *< .001)
Per 1 mm/m increase		1.138 (1.056–1.226, *p *= .001)	1.142 (1.031–1.265, *p *= .011)
LA diameter/BSA			
Bottom two tertiles	20 (5.8)	Ref	Ref
Top tertile	27 (17.3)	3.164 (1.773–5.646, *p *< .001)	2.671 (1.395–5.117, *p *= 0.003)
per 1 mm/m^2^ increase		1.110 (1.039–1.185, *p *= 0.002)	1.107 (1.004–1.221, *p *= 0.042)

CI, confidence interval; HR, hazard ratio; LA diameter/H, left atrial diameter/height; LA diameter/BSA, left atrial diameter/ body surface area.

Data are presented as number (recurrent ischemic stroke per 100 patient‐years).

Adjusted for age, sex, hypertension, diabetes mellitus, hypercholesterolemia, atrial fibrillation, smoking, LV ejection fraction, and the use of antiplatelet and anticoagulation agents.

### 3.3 Association between LA size and recurrent cardioembolic or cryptogenic stroke

**Figure 2:**
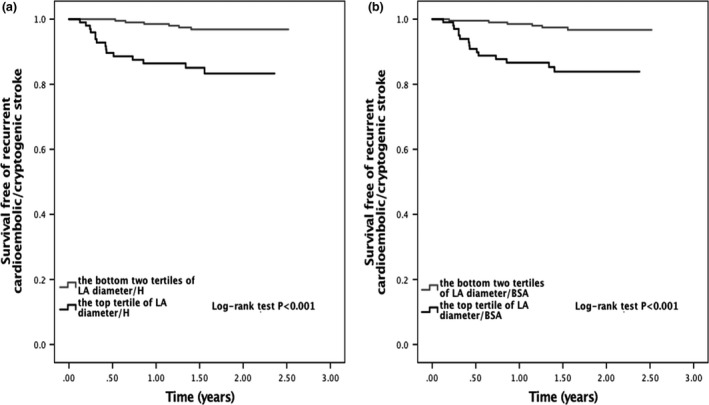
Survival curves free of recurrent cardioembolic/cryptogenic stroke according to the left atrial diameter/height (a) and left atrial diameter/body surface area (b)

Among the 47 ischemic stroke recurrence, 19 were large artery atherosclerosis (40.4%), 14 cardioembolic (29.8%), three lacunar (6.4%), and seven cryptogenic (14.9%). In the unadjusted model, LA diameter/H was associated with an increased risk of recurrent cardioembolic or cryptogenic ischemic stroke (unadjusted HR 6.023, 95% CI 2.336–15.532, *p *<* *.001). This relationship persisted after adjusting for age, sex, hypertension, diabetes mellitus, hypercholesterolemia, atrial fibrillation, smoking, LV ejection fraction, and the use of antiplatelet and anticoagulation agents (adjusted HR 5.673, 95% CI 1.780–18.084, *p *=* *.003; Table 4). As a continuous variable, LA diameter/H was also associated with recurrent cardioembolic or cryptogenic ischemic stroke (unadjusted HR 1.264 per 1 mm/m change in LA diameter/H, 95% CI 1.146–1.394, *p *< .001; adjusted HR 1.199 per 1 mm/m change in LA diameter/H, 95% CI 1.039–1.383, *p *= .013). In a secondary analysis, using LA diameter/BSA as continuous variable, the association attenuated in the multivariable model after adjustment (adjusted HR 1.113 per 1 mm/m^2^ change in LA diameter/BSA, 95% CI 0.975–1.271, *p *= .113). Kaplan–Meier survival analysis is shown in Figure 2.

**Table 4:** Association between LA size and recurrent cardioembolic or cryptogenic stroke

Left atrial size	No. of recurrent cardioembolic or cryptogenic ischemic stroke	Unadjusted HR	Adjusted HR
LA diameter/H			
Bottom two tertiles	6 (1.7)	Ref	Ref
Top tertile	15 (9.6)	6.023 (2.336–15.532, *p *< 0.001)	5.673 (1.780–18.084, *p *= 0.003)
Per 1 mm/m increase		1.264 (1.146–1.394, *p *< 0.001)	1.199 (1.039–1.383, *p *= 0.013)
LA diameter/BSA			
Bottom two tertiles	6 (1.7)	Ref	Ref
Top tertile	15 (9.6)	5.819 (2.256–15.009, *p *< 0.001)	3.095 (1.037–9.236, *p *= 0.043)
Per 1 mm/m^2^ increase		1.188 (1.097–1.287, *p *< 0.001)	1.113 (0.975–1.271, *p *= 0.113)

CI, confidence interval; HR, hazard ratio; LA diameter/H, left atrial diameter/height; LA diameter/BSA, left atrial diameter/ body surface area.

Data are presented as number (recurrent ischemic stroke per 100 patient‐years).

Adjusted for age, sex, hypertension, diabetes mellitus, hypercholesterolemia, atrial fibrillation, smoking, LV ejection fraction, and the use of antiplatelet and anticoagulation agents.

## 4 Discussion

Our study showed that, in a population of initial ischemic stroke patients in China, LA diameter/H was significantly associated with the total recurrent ischemic stroke and the composite of recurrent cardioembolic or cryptogenic stroke over a follow‐up period of 1.63 years. This association persisted even after adjusting for baseline demographics, clinical risk factors, echocardiographic LV ejection fraction, and medication. However, in a secondary analysis, LA diameter/BSA as a continuous variable was not associated with the composite of recurrent cardioembolic or cryptogenic stroke in a multivariable model, suggesting that LA diameter/H was a stronger predictor of stroke recurrence than LA diameter/BSA.

A number of studies have evaluated the relationship between LA size and stroke based on the general population. Barnes et al. (2004) found that LA volume was independently predictive of first ischemic stroke in an elderly cohort with no prior AF. Nagarajarao et al. (2008) reported that LA size, as evaluated by LA diameter/H, was a predictor of stroke, but the relation attenuated after adjustment for LV hypertrophy and LV ejection fraction. On the contrary, LA diameter was not found to be associated with stroke in two prospective studies (Gardin et al., 2001; Kizer et al., 2006). In addition, Benjamin et al. (1995) and Di Tullio et al. (1999) reported that LA size was a predictor of ischemic stroke only in men, whereas Bouzas‐Mosquera et al. (2011) found a relationship between LA diameter and ischemic stroke only in women. The different characteristics of enrolled subjects and different measurements for LA size might explain some of these discrepancies.

In this study, we focused on the predictive value of LA size for recurrent ischemic stroke and the probable embolic stroke (cardioembolic and cryptogenic stroke). To our knowledge, the relationship between LA size and recurrent ischemic stroke has been evaluated in two prospective studies (Paciaroni et al., 2015; Yaghi et al., 2015). In the Northern Manhattan Stroke Study, investigators found that moderate to severe LA enlargement was an independent marker of recurrent cardioembolic or cryptogenic stroke in a multiethnic cohort of ischemic stroke patients, suggesting that these two subtypes may share a common embolic mechanism (Yaghi et al., 2015). Our study showed that elevated LA size was not only associated with the embolic stroke (cardioembolic and cryptogenic stroke), but also with the total recurrent ischemic stroke. In the RAF study, investigators also found that LA enlargement was an independent marker of recurrent stroke and systemic embolism within 90 days in patients with AF‐associated acute stroke (Paciaroni et al., 2015). Recently, several studies have focused on the role of electrocardiographic LA abnormality for predicting stroke. In the Atherosclerosis Risk In Communities Study, advanced inter‐atrial block was found to be correlated with incident ischemic stroke, prompting that left atrial disease should be considered as an independent stroke risk factor (O’ Neal et al., 2016). Electrocardiographic P‐wave terminal force in lead V1 was reported to be associated with total recurrent ischemic stroke and the composite of cryptogenic or cardioembolic stroke independent of AF, suggesting a specific link with left atrial thromboembolism (Kamel et al., 2015). Similarly, our study found that echocardiographic LA dilatation was associated with increased risk of recurrent ischemic stroke and the combined outcome of cardioembolic and cryptogenic stroke.

Several reasons could be proposed to explain the association of LA enlargement with recurrent ischemic stroke. One potential mechanism is blood stasis, and thrombus formation might occur more frequently as left atrial size increase (Benjamin et al., 1995). It has been shown that with an increase in left atrial volume, reduced flow velocity in the left atrial appendage results in an increased risk for thrombus formation and potential for embolic stroke (Benjamin et al., 1995; Cujec, Polasek, Voll, & Shuaib, 1991; Daniel et al., 1988; Di Tullio et al., 1999). The thrombogenicity of LA enlargement has been supported by transesophageal echocardiographic studies, suggesting an association between LA dilatation and spontaneous echocardiographic contrast, LA thrombus, and embolic events (Benjamin et al., 1995). Another proposed mechanism is that LA enlargement serves as a strong risk factor for the development of atrial fibrillation, a well‐established condition that increases the risk of stroke (Benjamin et al., 1998; Di Tullio et al., 1999). Our results showed that LA enlargement remained an independent predictor of recurrent ischemic stroke even after adjusting for the history of AF. However, long‐term heart‐rhythm monitoring, which was proved to increase the detection of paroxysmal AF (Gladstone et al., 2014; Rizos et al., 2012), was not routinely performed in our study. As a result, we cannot rule out subsequent AF that could also explain our outcome. However, none of the patients who had recurrent stroke were found to have AF during follow‐up. Additionally, LA enlargement may serve as a marker for structural heart disease and hypertension, which increases the risk of recurrent stroke. It was reported that the relationship between LA size and incident stroke was lessen after adjusting for LV hypertrophy and LV ejection fraction (Nagarajarao et al., 2008). However, our multivariate analysis showed that the association between LA enlargement and recurrent ischemic stroke was independent of hypertension and LV ejection fraction. Finally, LA enlargement may serve as a surrogate for other unidentified risk factors for the incidence of recurrent ischemic stroke (Benjamin et al., 1995).

A number of limitations in this study should be considered. First, we did not monitor our patients for atrial fibrillation during the period of follow‐up visits, so the potential AF status may be missed. Second, there was insufficient power to estimate the relationship with recurrent stroke in different degrees of LA enlargement due to the small study population. Third, our study did not include measurement for LA volume, which is thought to be a more accurate measurement for left atrial size and also a stronger predictor of cardiovascular events (Khankirawatana, Khankirawatana, & Porter, 2004; Lemire, Tajik, & Hagler, 1976; Lester, Ryan, Schiller, & Foster, 1999).

In conclusion, this is the first study analyzing the association between LA size and recurrent ischemic stroke based on a Chinese population. We found that LA size is an independent predictor of total recurrent ischemic stroke and the composite of recurrent cardioembolic or cryptogenic stroke after adjusting for baseline demographics characteristics, clinical factors, echocardiographic LV ejection fraction, and medication.

## Conflict of Interest

The authors declare that they have no conflict of interests.
